# Spatial risk for gender-specific adult mortality in an area of southern China

**DOI:** 10.1186/1476-072X-6-31

**Published:** 2007-07-24

**Authors:** Mohammad Ali, Yang Jin, Deok Ryun Kim, Zhou Bao De, Jin Kyung Park, Rion Leon Ochiai, Baiqing Dong, John D Clemens, Camilo J Acosta

**Affiliations:** 1 International Vaccine Institute, Seoul, Korea; 2 Guangxi Centers for Disease Control and Prevention, Guangxi, China

## Abstract

**Background:**

Although economic reforms have brought significant benefits, including improved health care to many Chinese people, accessibility to improved care has not been distributed evenly throughout Chinese society. Also, the effects of the uneven distribution of improved healthcare are not clearly understood. Evidence suggests that mortality is an indicator for evaluating accessibility to improved health care services. We constructed spatially smoothed risk maps for gender-specific adult mortality in an area of southern China comprising both urban and rural areas and identified ecological factors of gender-specific mortality across societies.

**Results:**

The study analyzed the data of the Hechi Prefecture in southern in China. An average of 124,204 people lived in the area during the study period (2002–2004). Individual level data for 2002–2004 were grouped using identical rectangular cells (regular lattice) of 0.25 km^2^. Poisson regression was fitted to the group level data to identify gender-specific ecological factors of adult (ages 15–<45 years) mortality. Adult male mortality was more than two-fold higher than adult female mortality. Adults were likely to die of injury, poisoning, or trauma. Significantly more deaths were observed in poor areas than in areas with higher incomes. Specifically, higher spatial risk for adult male mortality was clustered in two rural study areas, which did not overlap with neighborhoods with higher risk for adult female mortality. One high-risk neighborhood for adult female mortality was in a poor urban area.

**Conclusion:**

We found a disparity in mortality rates between rural and urban areas in the study area in southern China, especially for adult men. There were also differences in mortality rates between poorer and wealthy populations in both rural and urban areas, which may in part reflect differences in health care quality. Spatial influences upon adult male versus adult female mortality difference underscore the need for more research on gender-related influences on adult mortality in China.

## Background

Although economic reforms have brought significant benefits, including improved health care to many Chinese people, the quality of care remains uneven. Higher quality health care is available mainly in cities [1]. There are also signs that for China's women, particularly in the countryside, the reform era has been associated with declining access to quality health care services, suggesting that inequalities in improved health care occur along gender and geographic boundaries [1]. The Chinese government has articulated its commitment to closing the gaps to accessing improved health care services. However, there is no clear understanding of the effects of health care quality by gender or socioeconomic status. It is, therefore, important to identify the variations in accessing improved health care services among different levels of society in order to understand the causes and the magnitude of problem. Some studies suggest that mortality is an important indicator for evaluating variations in accessibility to quality health care services [2,3].

Twigg *et al*. [4] postulated that human health-related behavioral practices are influenced by others within a society. Thus, addressing the impact of social ecology on health is important but greatly limited by available analytical tools. The lack of an effective geocomputational environment and algorithms has hindered the development of spatial analysis techniques [5] leading to unrealistic assumptions about human behavior. Related analytical problems include the uneven distribution of physical facilities, differences in speed of movement in different areas, and the effect of communications networks. To address these issues, geographical epidemiologists are increasingly using more complex methods of statistical analysis to investigate the spatial distribution of diseases [6,7].

Disease maps (spatial distribution of disease) provide researchers with visual displays that can suggest, via patterns of physical facilities and the human environment, useful avenues of research into causal processes [8,9]. However, the use of simple relative risk assessment of a disease (e.g., number of observed cases divided by number of expected cases for each area) may create problems in areas with small populations (usually rural areas), yielding extreme relative risks as the number of expected cases in the denominator is low. Methods for obtaining stable and accurate estimated rates in small populations are critical for effective analysis. Bayesian hierarchical modeling approach that uses Markov chain Monte Carlo (MCMC) procedure deals with complicated data structures and models and has good properties for a broad range of true underlying parameter arrangements [9].

Here we describe the use of neighborhood level data with identical rectangular 0.25-km^2 ^cells (regular lattice) in a Bayesian hierarchical modeling approach. We used an MCMC computational method to obtain the joint posterior distribution of model parameters, from which we constructed smoothed risk maps of gender-specific adult mortality in an area of southern China with both urban and rural areas. Significant gender imbalance in mortality rates (male mortality more than double that of females of similar ages) induced us to conduct this gender-specific mortality study.

## Results

The average population of the study area during the study period (2002–2004) was 124,204, of which 72% lived in urban areas. In total 1,576 deaths were reported yielding an annual mortality rate of 3.21 per 1000 people. Annual mortality rates varied by age and gender, ranging from 0.30/1000 in boys <5 years old to 17.15/1000 for men ≥45 years (Table 1). Overall, the male mortality rate was 1.6 time higher (95% confidence interval = 1.46, 1.79) than the female mortality rate (Table 1). The mortality rates in each of the 3 years were significantly higher in the rural areas than that in the urban areas (Table 2). In the study area, about 35% of the households were headed by women. Overall mortality in female-headed households was 7.66/1000/year (715/93392), which was more than twice as high as in households headed by men (3.06; 861/279220). The difference was statistically significant (relative risk [RR] = 2.48; 95% confidence interval = 2.25, 2.74).

**Table 1:** Population and deaths by gender and age groups, Hechi Prefecture, 2002–2004, Guangxi, China.

	Male			Female			
		
Age group, years	Average population*	Total deaths^†^	rate/year/1000	Average population*	Total deaths^†^	rate/year/1000	Relative risk male vs. female (95% CI)
<5	3352	3	0.30	2795	7	0.83	0.36 (0.09, 1.38)
5–<15	8670	6	0.23	8136	4	0.16	1.41 (0.40, 4.99)
15–<45	35756	178	1.66	34511	67	0.65	2.56 (1.94, 3.39)
≥45	15604	803	17.15	15380	508	11.01	1.56 (1.40, 1.74)

Total	63382	990	5.21	60822	586	3.21	1.62 (1.46, 1.79)

NOTE. CI, confidence interval.

*average population, 2002–2004

^†^total deaths, 2002–2004

**Table 2:** Overall mortality rates by year and areas (urban vs. rural), Hechi Prefecture, Guangxi, China.

	Urban			Rural			
		
Year	Population	Deaths	Rate/1000	Population	Deaths	Rate/1000	Relative risk (rural vs. urban) (95% CI)
2002	70,631	318	4.26	32,477	184	5.67	1.26 (1.05–1.51)
2003	97,500	350	3.59	36,177	162	4.48	1.25 (1.04–1.50)
2004	97,076	370	3.81	34,751	192	5.53	1.45 (1.22–1.72)

Total	265,207	1038	3.89	103,405	538	5.23	1.35 (1.22–1.50)

In the study age group (15–<45 years), the male mortality was more than double the female mortality (RR = 2.56; 95% confidence interval = 1.94, 3.39); Table 1). We also derived the rates (male = 1.58/1000/year, female = 0.62/1000/year) from the MCMC model, which are similar to the rates derived from actual observations. Most of the deaths in this age group (as determined from verbal autopsy data) were due to external causes such as injury, poisoning, toxicosis, and trauma followed by neoplasm (Table 3). Because verbal autopsies were not initiated until mid-2002, the numbers of deaths differed from the census data.

**Table 3:** Causes of death for men and women aged 15–<45 years, Hechi Prefecture, Guangxi, China, 2002–2004.

	Male		Female	
	
Cause of death	# of deaths	% of total deaths	# of deaths	% of total deaths
Diseases of blood and blood-forming organs and certain immune disorders	0	0.00	1	1.82
Diseases of the nervous system	0	0.00	2	3.64
Diseases of the genitourinary system	0	0.00	2	3.64
Pregnancy, childbirth, puerperium	0	0.00	1	1.82
Mental and behavioral disorders	1	0.71	0	0.00
Diseases of the ear and mastoid process	1	0.71	0	0.00
Diseases of the musculoskeletal system and connective tissue	1	0.71	1	1.82
Certain infectious and parasitic diseases	3	2.13	1	1.82
Endocrine, nutritional and metabolic diseases	4	2.84	0	0.00
Diseases of the respiratory system	4	2.84	0	0.00
Diseases of the circulatory system	10	7.09	4	7.27
Diseases of the digestive system	14	9.93	1	1.82
Abnormal symptoms, signs, clinical and laboratory findings	15	10.64	4	7.27
Neoplasms	21	14.89	16	29.09
External causes such as injuries, toxicosis, trauma	67	47.52	22	40.00

**Total**	**141**	**100**	**55**	**100**

### Aspatial regression model

The descriptive statistics showed no gender-specific variability in the covariates analyzed (Table 4). By univariate Poisson regression lower per capita neighborhood income, longer distance to hospital/health facility, and longer distance to the river were significantly associated with higher spatial risk for male mortality (data not shown). The multiple Poisson regression was fitted with all of the five variables listed in Table 4. The residuals of the multiple regression model were checked for spatial pattern by use of a Moran scatter plot. The results of this plot show no spatial patterns of the residuals (for males, Moran's I = -0.0024, p = .52; for female, Moran's I = -0.0194, p = .35) illustrating that necessary ecological variables were included in the model for predicting spatial risk of mortality.

**Table 4:** Study variables for neighborhoods (0.25-km^2 ^grid cells) for men and women aged 15 to <45 years.

	Men (n = 243 neighborhoods)				Women (n = 236 neighborhoods)			
	
Neighborhood variable	Mean	SD	Minimum	Maximum	Mean	SD	Minimum	Maximum
Per capita monthly income	288.29	254.79	24.16	2182	290.54	256.50	24.16	2182
Population density/km^2^	2,694	8,092	16	114,067	2,767	8,200	28	114,067
Hospital/health facility distance (km)	1.28	1.16	0.04	5.43	1.26	1.14	0.04	5.43
Distance from river (km)	1.00	1.03	0.00	4.83	0.98	1.00	0.00	4.48
Per capita health care* expenditure in last month of 2001 census^†^	15.33	54.81	0	810	15.74	55.57	0	810

NOTE. SD, standard deviation. Income and expenditures are in renminbi (1 USD = 7.75 RMB).

* Outpatient visits, physician fees, hospital costs, medicines, and lab tests.

^†^The last month data was collected during census 1 (late 2001).

By multiple regression analysis only lower per capita income was significantly associated with higher spatial risk for male mortality (Table 5). While the univariate regression model fitted for female mortality did not yield any significant variables associated with higher spatial risk of mortality (data not shown), multiple regression showed that lower per capita income was associated with higher spatial risk for female mortality after adjusting for all other variables in the model (Table 6). Higher per capita health care expenditure in the neighborhood had a significant positive association with higher spatial risk for female mortality in the multiple regression model.

**Table 5:** Mortality for men aged 15 to <45 years by multiple Poisson regression, 2002–2004, Hechi Prefecture, Guangxi, China.

Parameter	Estimate	Standard Error	Wald 95% Confidence Limits	χ^2^	Pr > χ^2^
Per capita monthly neighborhood income	-0.0014	0.0006	-0.0025, -0.0002	5.06	0.0245
Neighborhood population density//km^2^	-0.0000	0.0000	-0.0000, 0.0000	0.05	0.8293
Hospital/health facility distance (km)	-0.0312	0.1430	-0.3114, 0.2491	0.05	0.8275
Distance from river (km)	0.2413	0.1420	-0.0371, 0.5197	2.89	0.0893
Per capita neighborhood health care expenditure in the last month of 2001 census	0.0042	0.0031	-0.0019, 0.0102	1.83	0.1763

NOTE. Income and expenditures are in renminbi (1 USD = 7.75 RMB).

**Table 6:** Mortality for women aged 15 to <45 years by multiple Poisson regression, 2002–2004, Hechi Prefecture, Guangxi, China.

Parameter	Estimate	Standard Error	Wald 95% Confidence Limits	χ^2^	Pr > χ^2^
Per capita monthly neighborhood income	-0.0020	0.0010	-0.0040, -0.0001	4.05	0.0442
Neighborhood population density//km^2^	0.0000	0.0000	-0.0000, 0.0000	1.65	0.1987
Hospital/health facility distance (km)	-0.0592	0.2587	-0.5664, 0.4479	0.05	0.8189
Distance from river (km)	0.0894	0.2565	-0.4134, 0.5923	0.12	0.7274
Per capita neighborhood health care expenditure in last month of the 2001 census	0.0057	0.0023	0.0012, 0.0103	6.09	0.0136

NOTE. Income and expenditures are in renminbi (1 USD = 7.75 RMB).

### Spatial model

In the MMMC model, we used both fixed and random effects of the neighborhood and adjoining neighborhoods and fixed effect of the per capita neighborhood income. The parameter estimates of the models fitted for adult male and female mortalities are presented in Table 7. The MMMC model yielded neighborhood per capita income as an influencing factor for predicting higher risk for adult male mortality, but not for female mortality. We also checked the results using the CAR model (results not shown), which did not show any improvement over the multiple-membership model as obtained from the Bayesian Deviation Information Criterion (DIC) diagnostics [10]; thus we confined our spatial risk analysis using the multiple-membership model.

**Table 7:** Parameter estimates by a multiple-membership multiple classification model for men and women aged 15 to <45 years.

	Men				Women			
	
Parameter	Estimate	SE	χ^2^	Pr>χ^2^	Estimate	SE	χ^2^	Pr>χ^2^
Intercept	0.482	0.179	7.267	.007	0.379	0.323	1.372	.241
Per capita monthly neighborhood income (in RMB)	-0.001	0.000	9.299	.002	-.001	0.000	2.295	.129
Level 2 residual variance	0.032	0.046	0.480	.488	0.014	0.022	0.415	.519
Level 3 residual variance	0.127	0.207	0.373	.541	0.287	0.658	0.191	.662
Deviance (MCMC)	421.204 (243 of 243 cases in use)				229.999 (236 of 236 cases in use)			

NOTE. MCMC, Markov chain Monte Carlo computational method; RMB, renminbi

### Mortality mapping

**Figure 1:**
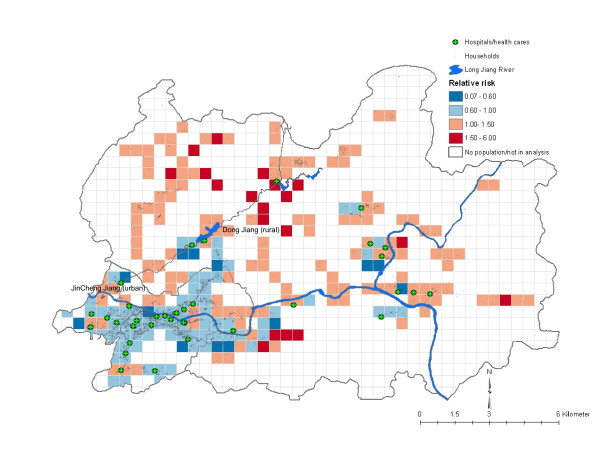
**Spatial variations of relative risk (see text) for adult (15–<45 years) male mortality, Hechi Prefecture, Guangxi, China, 2002–2004**. The spatial variations of relative risks from lower to higher are shown in diverging color scheme (blue to red) in ColorBrewer.

**Figure 2:**
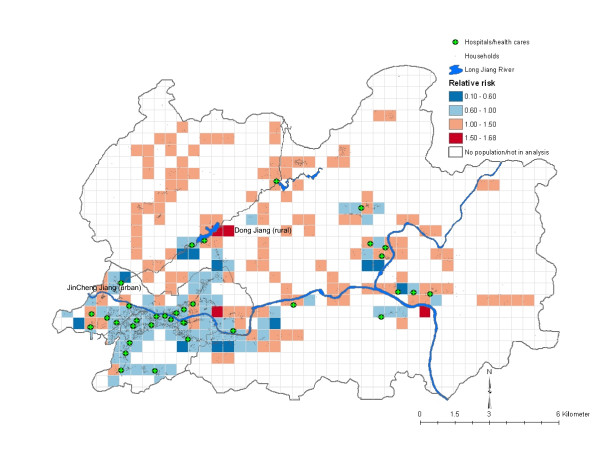
**Spatial variations of relative risk (see text) for adult (15–<45 years) female mortality, Hechi Prefecture, Guangxi, China, 2002–2004**. The spatial variations of relative risks from lower to higher are shown in diverging color scheme (blue to red) in ColorBrewer.

The mapping of adult mortality in terms of spatially smoothed relative risk (exponentiation of the predicted estimate obtained from the MMMC model) is shown in Figure 1. Higher spatial risk (RR, ≥ 1.5) for adult male mortality was found only in rural part of the study area. Two regional clusters can be derived for the high-risk neighborhoods from this map. Yet, even though adult female mortality was higher in more rural than urban neighborhoods (3 vs. 1), no spatial patterns for high-risk female mortality are shown on the map (Figure 2). There were far more high-risk neighborhoods for adult male mortality than for adult female mortality (19 vs. 4) and none were superimposed. The only high-risk urban neighborhood showed an increase in female mortality. This area was very poor (per capita income, 158 RMB vs. 290 RMB for all neighborhoods).

## Discussion

In our study, adult mortality was significantly higher for males than that for females. Adults were likely to die of injury, poisoning, or trauma. Overall mortality was higher in households headed by women. The disparity in the mortality rates between adult men and women suggests that if this trend continues, there will be more households with women heads, which could eventually increase the number of deaths in the study area. Our results also illustrate disparity in mortality rates between rural and urban areas, which may in part be due to disparities in health care accessibility.

In our aspatial analysis, the risk for adult mortality was higher in impoverished communities (defined by lower per capita neighborhood monthly income) than in wealthier communities, suggesting that the benefits of improved health care were not evenly distributed throughout the study area. The collapse of China's Cooperative Medical System in 1978 resulted in the lack of an organized financing scheme for health care, adversely affecting access by rural people to health care, especially the poor [11]. This could perhaps account for the high number of "at risk" neighborhoods in the impoverished part of our rural study area. The poor urban neighborhood at high risk for female mortality suggests that even in areas where health care is easier to access because it is closer by, gender influences who receives health care services in poor societies.

The adult mortality maps (Figures 1 and 2) show many neighborhoods with increased risks of mortality (RR, >1.5). These neighborhoods formed two regional clusters, both in the rural area. In contrast, not many neighborhoods carried increased risk for adult female mortality. These maps may suggest difference in adverse effects of health hazards across subpopulations [9]. Because none of the high-risk neighborhoods for male and female mortality were superimposed, there is a possibility that adult men and women in southern China face different ecological and environmental risks. Future studies should analyze risk factors in greater depth.

We found significantly higher mortality in rural than in urban areas, possibly because of less health care accessibility [1]. Also, health services available in rural areas may not provide adequate treatment for potentially curable diseases. Chinese policymakers are currently trying to narrow the disparity in health care services for rural and urban residents through a five-part reform policy. Specific targets are directed at (1) increasing public funding for primary health care; (2) providing quality and accessible health care; (3) extending coverage of social health insurance schemes; (4) providing government health subsidies to vulnerable portions of the population; and (5) adopting more appropriate health technologies and pharmaceuticals in health care delivery.

A potential limitation of our study is the arbitrary choice of neighborhoods and therefore variations in the size of population across neighborhoods. We believe our selection is an appropriate compromise between loss of resolution and excess dispersion. One may argue that the mass vaccine campaign might have influenced neighborhood level variations in mortality rates. However, the trial was cluster randomized and we assumed that ecological determinants were independent of the cluster effects under the trial design for vaccine assignment. Moreover, the rates of infections targeted by the vaccines were too low to have affected overall mortality. Another limitation of our study design is that our study area has an urban area with a high population density and a sparsely populated rural area. Thus, the population varied greatly across neighborhoods. However, since we incorporated the neighboring area effect into the model, we believe the model adequately addressed heterogeneity of across-neighborhood population. Because we assigned equal weights for neighborhood components without knowing the spatial influences of mortalities and/or anisotropy on the surface, complexities may have been diminished.

Although both the models (MMMC and CAR) can be used to account for the effects of locations, there are differences between the two models. In MMMC model, we consider two sets of random effects: exchangeable area random effects and a multiple membership set of random effects for the neighbors of each neighborhood. This mean the rates in each neighborhood is affected by both the neighborhood and its nearby neighbors. The weight columns contain equal weights for each neighboring neighborhood that sum to 1. In contrast, the CAR prior is a spatial smoothing prior, and individual random effect is not random in the CAR model. This model has only one random effect for each neighborhood, and it is expected value the average of surrounding random effect. Note to make the CAR model identifiable we either need to constrain random effect to sum to 0 or remove the intercept from the model. CAR procedure typically uses weight of 1 for all observations, as these weights will then be divided by the number of neighbors in the model.

We focused on the production of reliable maps for gender-specific adult mortality in an area of southern China. By using the Bayesian hierarchical model, the neighborhood random effects were posterior sampled and the associated relative risk estimates were averaged to produce a posterior-average relative risk [12], which was then used to produce the gender-specific mortality maps. The approach produces stable and accurate estimates so that data modeling with this approach implies greater reliability in identifying areas at greatest risk for mortality and the underlying reasons.

## Methods

### Study area and data

The study was conducted in the Hechi Prefecture of Guangxi Zhuang Autonomous Region (Guangxi Province) in southern China, which borders Guangdong Province in the east and Vietnam in the south. Hechi Prefecture is in the northwest of the province, approximately 400 km from the provincial capital, Nanning. The catchment area includes two populous areas: Jin Cheng Jiang, an urban area (26 km^2^), and Don Jiang, a rural area (191 km^2^). The site was originally set up by the International Vaccine Institute (IVI) in collaboration with the Guangxi Centers for Disease Control and Prevention for a multi-centric Vi polysaccharide vaccine effectiveness evaluation for typhoid fever [13,14]. All residents of Jin Cheng Jiang and Dong Jiang were enumerated in late 2001 by the vaccine trial project staff and were followed in subsequent years for medical and vital demographic events including deaths. According to the project's census, an average of 124,204 people lived in the area, half male. Beginning in mid-2002 all deaths in the community were recorded in yearly census surveys conducted by project staff.

The project's mortality surveillance team collected information from sentinel posts (i.e., hospitals, funeral houses, family planning offices and local government, village, and police registrars). A modified (shorter) verbal autopsy (based on procedures developed by the UK Department for International Development, 1997; the World Health Organization; The Johns Hopkins School of Hygiene and Public Health; and The London School of Hygiene and Tropical Medicine, 1999) was used by a trained physician to determine cause of death. The verbal autopsy form containing data on the cause of death (using locally adapted classifications of diseases) was entered into the project database. The verbal autopsy was conducted only for the vaccine trial target population (ages 5–60 years).

We created a household geographic information system (GIS) in the study area using handheld global positioning system receivers. Among the geographic features included in the GIS were Long Jiang River and its branches, roads, and mountains plus the 35 hospitals/health facility from which disease surveillance was carried out. By linking demographic and mortality data, the GIS allowed us to pursue spatial modeling of adult mortality in the study area.

### Data aggregation

We divided the study area into grid cells of 500 × 500 m, which we called neighborhoods. Grid cells less than 0.25 km^2 ^resulted in overdispersion of the mortality data (that is greater variation in the data than expected while larger cells obscured details of the ecological status) [15]. We removed parts of cells that fell outside the study area boundary. In total, we obtained 267 neighborhoods with at least one person living in the neighborhood. We subsequently excluded neighborhoods with fewer than four persons or no neighboring cell with at least four persons because neighborhood level ecological data derived from few observations could bias the outcome. Ultimately we obtained 243 neighborhoods with at least four adult males (ages 15–<45 years) in each and 236 neighborhoods with at least four adult females (ages 15–<45 years). Mortality data for individuals and several socioeconomic covariates were aggregated by neighborhood. Linear distances to the nearest hospital/health facility and to the nearest river side were computed from the neighborhood center.

### Poisson regression analysis

We fitted a Poisson regression model to analyze neighborhood level mortality data. In a Poisson regression model, observed counts are assumed to have Poisson distribution, with expected values depending on *p *predictor variables ***x ***= (*x*_1_, *x*_2_,..., *x*_*p*_)^*T*^. We used the following model for our analysis:

*Log *[*E*(*Y*)] = *log*(*exp*) + *β*_0 _+ *β*_1_*x*_1 _+ ...... + *β*_5_*x*_5_,

a generalized linear model with log link function and Poisson distributed errors where *E*(*Y*) is the expectation of observation, *log*(*exp*) is the logarithm of expected number of cases, *x*_1 _is monthly per capita neighborhood income, *x*_2 _is population density in the neighborhood, *x*_3 _is hospital/health facility distance, *x*_4 _is distance from the river, and *x*_5 _is neighborhood per capita expenditure on health care in a month (considered the last month of the census 1 survey conducted in late 2001). *βi *is the coefficient corresponding to *x*_*i*_. The term *log*(*exp*) was an offset with the parameter estimate constrained to 1 since we were interested in (relative) rates rather than counts. Neighborhood income is a surrogate for the neighborhood economic status, population density and distance from river are the surrogates of environmental differences among neighborhoods, distance to hospital/health facility describes access to health care, and health care expenditure is a surrogate for health care utilization. We used Stata/SE 9.0 for Windows (StataCorp LP, College Station, TX 77845 USA) to analyze the data using Poisson regression.

### Univariate Moran scatter plot

To assess spatial patterns we analyzed the residuals of the Poisson multiple regression model using univariate Moran scatter plot (GeoDA™ software; Luc Anselin and the Regents of the University of Illinois). The spatial weight was determined using first order Queen Contiguity (i.e., all common points including boundaries and vertices were included in the neighbor definition). The method produces four quadrants within the graph that provides a classification of two types of positive spatial autocorrelation: *high-high *(upper right), *low-low *(lower left); and two types for negative spatial autocorrelation: *high-low *(lower right) and *low-high *(upper right). Inference for Moran's I was based on a permutation approach, in which a reference distribution is calculated for spatially random layouts with the same data as observed. The randomization uses an algorithm to generate spatially random simulated data sets as outlined by Anselin [16]. We used 9999 random permutations in constructing the reference distribution.

### Spatial model

The standard hierarchical model structure does not explicitly incorporate spatial structure, although through the use of higher levels of geography as additional levels in the model, we can indirectly incorporate spatial clustering effects. One extension to the standard hierarchical model is the multiple-membership model that addresses the effect of spatial correlation between neighboring areas [17]. Browne *et al. *[18] consider Bayesian extensions of this model as a member of the family models they call a multiple-membership multiple classification (MMMC) model. Alternatively, we can use a conditional autoregressive (CAR) model that also assesses spatial correlation between neighboring areas. We used MLwiN Version 2.0 (^©^Multilevel Models Project, Center for Multilevel Modelling, University of Bristol, UK) to fit MMMC models; for CAR models we used through likelihood-based estimation methods and MCMC estimations.

The spatial model considers observed counts for a set of areas with a known neighborhood structure. Thus, in the model specification the observed count is affected by the area where the count is taken and the neighboring areas. The approach considers a three classification model that includes the "identity" classification for the lowest level, a single membership classification, and a multiple member classification. The Poisson MMMC model is defined as

*y*_*i *_~ *Poisson*(*π*_*i*_)

log⁡(πi)=Xiβ+Zi(2)uC2(i)(2)+∑j∈C3(i)wi,j(3)Zi(3)uj(3)
 MathType@MTEF@5@5@+=feaafiart1ev1aaatCvAUfKttLearuWrP9MDH5MBPbIqV92AaeXatLxBI9gBaebbnrfifHhDYfgasaacH8akY=wiFfYdH8Gipec8Eeeu0xXdbba9frFj0=OqFfea0dXdd9vqai=hGuQ8kuc9pgc9s8qqaq=dirpe0xb9q8qiLsFr0=vr0=vr0dc8meaabaqaciaacaGaaeqabaqabeGadaaakeaacyGGSbaBcqGGVbWBcqGGNbWzcqGGOaakiiGacqWFapaCdaWgaaWcbaGaemyAaKgabeaakiabcMcaPiabg2da9iabdIfaynaaBaaaleaacqWGPbqAaeqaaOGae8NSdiMaey4kaSIaemOwaO1aa0baaSqaaiabdMgaPbqaaiabcIcaOiabikdaYiabcMcaPaaakiabdwha1naaDaaaleaacqWGdbWqdaWgaaadbaGaeGOmaidabeaaliabcIcaOiabdMgaPjabcMcaPaqaaiabcIcaOiabikdaYiabcMcaPaaakiabgUcaRmaaqafabaGaem4DaC3aa0baaSqaaiabdMgaPjabcYcaSiabdQgaQbqaaiabcIcaOiabiodaZiabcMcaPaaakiabdQfaAnaaDaaaleaacqWGPbqAaeaacqGGOaakcqaIZaWmcqGGPaqkaaGccqWG1bqDdaqhaaWcbaGaemOAaOgabaGaeiikaGIaeG4mamJaeiykaKcaaaqaaiabdQgaQjabgIGiolabdoeadnaaBaaameaacqaIZaWmaeqaaSGaeiikaGIaemyAaKMaeiykaKcabeqdcqGHris5aaaa@694D@

uC2(i)(2)~N(0,∑u(2))anduj(3)=N(0,∑u(3)).
 MathType@MTEF@5@5@+=feaafiart1ev1aaatCvAUfKttLearuWrP9MDH5MBPbIqV92AaeXatLxBI9gBaebbnrfifHhDYfgasaacH8akY=wiFfYdH8Gipec8Eeeu0xXdbba9frFj0=OqFfea0dXdd9vqai=hGuQ8kuc9pgc9s8qqaq=dirpe0xb9q8qiLsFr0=vr0=vr0dc8meaabaqaciaacaGaaeqabaqabeGadaaakeaafaqabeqadaaabaGaemyDau3aa0baaSqaaiabdoeadnaaBaaameaacqaIYaGmaeqaaSGaeiikaGIaemyAaKMaeiykaKcabaGaeiikaGIaeGOmaiJaeiykaKcaaOGaeiOFa4NaemOta4KaeiikaGIaeGimaaJaeiilaWYaaabeaeaacqGGPaqkaSqaaiabdwha1jabcIcaOiabikdaYiabcMcaPaqab0GaeyyeIuoaaOqaaiabbggaHjabb6gaUjabbsgaKbqaaiabdwha1naaDaaaleaacqWGQbGAaeaacqGGOaakcqaIZaWmcqGGPaqkaaGccqGH9aqpcqWGobGtcqGGOaakcqaIWaamcqGGSaaldaaeqaqaaiabcMcaPaWcbaGaemyDauNaeiikaGIaeG4mamJaeiykaKcabeqdcqGHris5aaaakiabc6caUaaa@58B9@

Here *y *is an N (number of lowest level unit) vector, *β *is a vector of fixed effect parameters, and ui(2)
 MathType@MTEF@5@5@+=feaafiart1ev1aaatCvAUfKttLearuWrP9MDH5MBPbIqV92AaeXatLxBI9gBaebbnrfifHhDYfgasaacH8akY=wiFfYdH8Gipec8Eeeu0xXdbba9frFj0=OqFfea0dXdd9vqai=hGuQ8kuc9pgc9s8qqaq=dirpe0xb9q8qiLsFr0=vr0=vr0dc8meaabaqaciaacaGaaeqabaqabeGadaaakeaacqWG1bqDdaqhaaWcbaGaemyAaKgabaGaeiikaGIaeGOmaiJaeiykaKcaaaaa@324B@, uj(3)
 MathType@MTEF@5@5@+=feaafiart1ev1aaatCvAUfKttLearuWrP9MDH5MBPbIqV92AaeXatLxBI9gBaebbnrfifHhDYfgasaacH8akY=wiFfYdH8Gipec8Eeeu0xXdbba9frFj0=OqFfea0dXdd9vqai=hGuQ8kuc9pgc9s8qqaq=dirpe0xb9q8qiLsFr0=vr0=vr0dc8meaabaqaciaacaGaaeqabaqabeGadaaakeaacqWG1bqDdaqhaaWcbaGaemOAaOgabaGaeiikaGIaeG4mamJaeiykaKcaaaaa@324F@ are the vectors of residuals for the random effects for classifications 2 and 3, respectively. Zi(2)
 MathType@MTEF@5@5@+=feaafiart1ev1aaatCvAUfKttLearuWrP9MDH5MBPbIqV92AaeXatLxBI9gBaebbnrfifHhDYfgasaacH8akY=wiFfYdH8Gipec8Eeeu0xXdbba9frFj0=OqFfea0dXdd9vqai=hGuQ8kuc9pgc9s8qqaq=dirpe0xb9q8qiLsFr0=vr0=vr0dc8meaabaqaciaacaGaaeqabaqabeGadaaakeaacqWGAbGwdaqhaaWcbaGaemyAaKgabaGaeiikaGIaeGOmaiJaeiykaKcaaaaa@3215@*and *Zi(3)
 MathType@MTEF@5@5@+=feaafiart1ev1aaatCvAUfKttLearuWrP9MDH5MBPbIqV92AaeXatLxBI9gBaebbnrfifHhDYfgasaacH8akY=wiFfYdH8Gipec8Eeeu0xXdbba9frFj0=OqFfea0dXdd9vqai=hGuQ8kuc9pgc9s8qqaq=dirpe0xb9q8qiLsFr0=vr0=vr0dc8meaabaqaciaacaGaaeqabaqabeGadaaakeaacqWGAbGwdaqhaaWcbaGaemyAaKgabaGaeiikaGIaeG4mamJaeiykaKcaaaaa@3217@ are the vectors of predictor values and wi,j(3)
 MathType@MTEF@5@5@+=feaafiart1ev1aaatCvAUfKttLearuWrP9MDH5MBPbIqV92AaeXatLxBI9gBaebbnrfifHhDYfgasaacH8akY=wiFfYdH8Gipec8Eeeu0xXdbba9frFj0=OqFfea0dXdd9vqai=hGuQ8kuc9pgc9s8qqaq=dirpe0xb9q8qiLsFr0=vr0=vr0dc8meaabaqaciaacaGaaeqabaqabeGadaaakeaacqWG3bWDdaqhaaWcbaGaemyAaKMaeiilaWIaemOAaOgabaGaeiikaGIaeG4mamJaeiykaKcaaaaa@348E@ is a scalar weight for the classification 3 unit *j *for lowest level unit *i*.

To fit the data into the model, we employed neighbors of the neighborhoods in level 3, and the neighborhood (group) in levels 1 and 2. We included a covariate "per capita neighborhood income," which had a significant relationship with mortality in the aspatial regression model, in the fixed part of the model (*Xβ*), in addition to the intercept term (CONS). The use of a covariate in a Bayesian spatial model is important for investigating environmental variations [9]. The CONS was a vector of 1s, which allowed for a variance component for each neighborhood to be estimated. The weight was employed based on the first-order neighborhood using Queen Contiguity (i.e., both boundaries and vertices are included in the definition). Hence we had eight neighbor columns and eight weight columns. For modeling, we fitted the variance component using a burn-in period of 500 and a chain of 50,000.

### Spatially smoothed relative risk of mortality

To evaluate the status of each neighborhood with respect to adult mortality, we obtained spatially smoothed relative risks of adult mortality within neighborhoods by means of the Bayesian approach described above. In general, the relative risk in disease/mortality mapping measures whether an area has a higher occurrence of disease incidence/mortality than that expected from the reference rate. In Bayesian disease/mortality models, the relative risk decomposes into two parts that are fixed terms consisting of overall level of relative risk and due to covariates and random terms. The random terms are spatial correlation structure, which introduces estimates of the risk in any area depending on neighboring areas, and uncorrelated heterogeneity, which pertains to the random sampling variability of the observed counts about the local mean.

## Competing interests

The author(s) declare that they have no competing interests.

## Authors' contributions

MA contributed to the design and analysis of the study and writing of the paper; YJ, RLO, CJA implemented the execution and supervision of the study, and writing of the paper; DRK contributed to the spatial analysis and writing of the paper; JKP managed the database and contributed to the analysis of the data; ZBD managed the database; BD and JDC were the principal investigators responsible for the drafting, adaptation, and implementation of the study protocol. All authors read and approved the final manuscript.
